# Considerations and concerns regarding the readiness to remove face coverings

**DOI:** 10.7189/jogh.12.03036

**Published:** 2022-06-21

**Authors:** Mari Terada, Shinya Tsuzuki, Yusuke Asai, Sho Saito, Norio Ohmagari

**Affiliations:** 1 Disease Control and Prevention Center, National Center for Global Health and Medicine, Tokyo, Japan; 2 Center for Clinical Sciences, National Center for Global Health and Medicine, Tokyo, Japan; 3 AMR Clinical Reference Center, National Center for Global Health and Medicine, Tokyo, Japan; 4 Faculty of Medicine and Health Sciences, University of Antwerp, Antwerp, Belgium

As of February 24, 2022, the government of the United Kingdom has taken a step towards deregulation and has lifted most of the COVID-19 related restrictions, including face-covering requirements in indoor public places [1,2]. People tested positive for COVID-19 were exempted from self-isolation and close contact tracing. The recent peak of the pandemic due to the Omicron variant seems to have passed in Europe, and the number of new cases in the UK is returning to pre-Omicron levels. Thus, it is a natural moment to alleviate COVID-19-related restrictions for the normalisation of economic activities. However, the question on the necessity of abolishing the requirement to wear face coverings remains. As WHO guidelines strongly recommend the use of face masks in public or when interacting with people outside the household [3], we doubt the hastiness of removing them – if they are not mandated, they should at least be strongly recommended. The number of COVID-19 cases in Japan has remained at a lower level than in European countries, and this may be attributed to the high rate of compliance with wearing face coverings among Japanese people.

**Figure 1** shows the low number of confirmed infections in Japan (3 492 799) compared to the UK (17 977 601) as of February 8, 2022 [4,5]. During the same period, the maximum number of cases per million people was still lower in Japan (827.801 on February 3, 2022) than in the UK (3515.061 on January 4, 2022) [5]. The reason for this is unclear, but it is notable that the rate of mask-wearing, which was high under all circumstances with no mandating policies, may have contributed to this. Using a smartphone-based native application “Kazereco”, we are investigating common cold symptoms and related behaviour among the application users. The app is publicly available on the App Store (Apple Inc., Cupertino CA, USA) and Google Play Store (Google LLC, Mountain View CA, USA) for anyone older than 16 years. Upon initially launching the app, users are asked to read through the study information sheet and give anonymous consent to participate in and submit data to the study. Using the app, respondents are asked to answer a daily questionnaire which includes the respondents’ subjective perception of the rate of mask-wearing when they are in public places (both indoors and outdoors). We calculated the daily mean on mask adherence rates from 2871 app users. The number of respondents on each day ranged from 48 to 273, as responses were voluntary and not all users used the application every day. The rate of daily mask adherence between January 16, 2021, and February 9, 2022, ranged from 86.4 to 99.8%. Notably, the rate remained high from October to December 2021, when the number of COVID-19 infections was particularly low in Japan.

**Figure:**
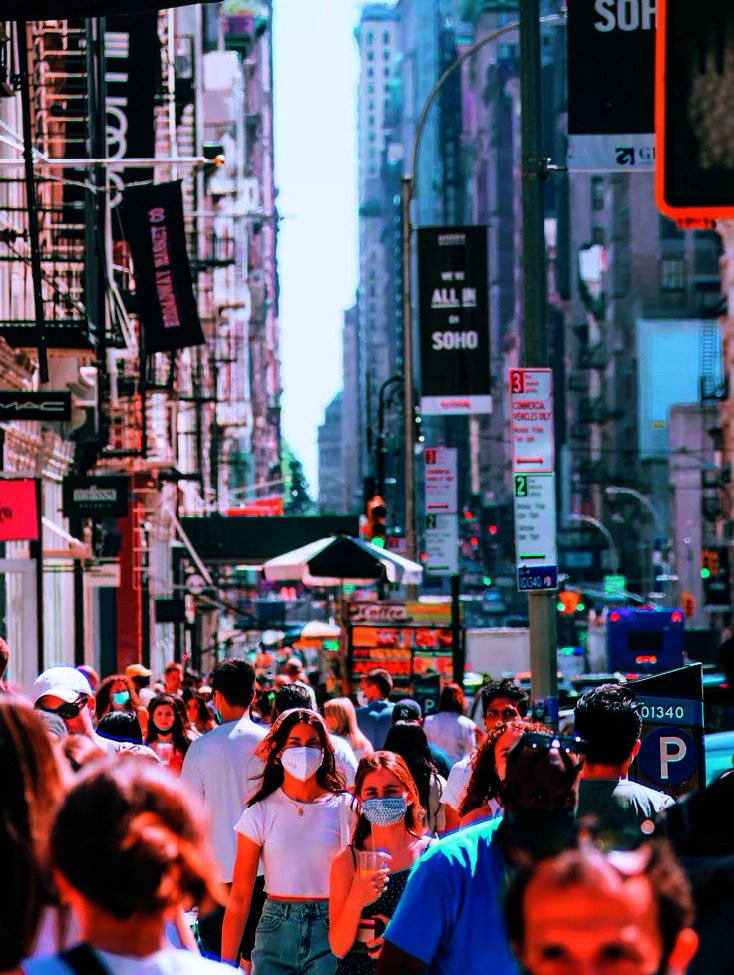
Photo: SoHo, Manhattan, New York. Source: Yoav Aziz, via https://unsplash.com/photos/T4ciXluAvIE. Free to use under the Unsplash license.

Evidence on mask-wearing may not be robust, but few will argue against the use of this simple and minimal measure in reducing the spread of the virus. In fact, a universal face-covering policy alone prevented the spread of COVID-19 from two hair salon stylists to their 139 clients in the US, whereas cohabitants of one of the stylists all eventually became infected [6]. A larger community study also implied that the introduction of a mask policy, mandating face coverage in all spaces outside the home, played a greater role to reduce infection growth rate than curfew or activity restrictions [7]. As these studies suggest, even under COVID-19 infection, mask-wearing may enable the continuation of normal business operations by preventing transmission.

As the COVID-19 pandemic continues, people have been forced to live a more restricted life. The de-regulation of COVID-19-related restrictions is encouraging news bringing hope that pre-pandemic life can be resumed. It has also been a great challenge to balance economic activities while controlling the COVID-19 pandemic. Governments are struggling against infection control and mitigation of COVID-19-related restrictions to resume economic activities. While lockdowns or activity restrictions negatively affect economies and prevent people from conducting normal business operations, socializing, travelling short or long distances, or other activities, mask-wearing does not. This is possibly why masking guidelines remain valid in many countries even after mandates are lifted [8,9]. The continued recommendation of mask-wearing in public places may be the best solution, as it does not interfere with economic activities. Further evidence on the use of masks, especially under no activity restriction, is needed.
